# Transformation of CuO Nanoparticles in the Aquatic Environment: Influence of pH, Electrolytes and Natural Organic Matter

**DOI:** 10.3390/nano7100326

**Published:** 2017-10-14

**Authors:** Cheng Peng, Chensi Shen, Siyuan Zheng, Weiling Yang, Hang Hu, Jianshe Liu, Jiyan Shi

**Affiliations:** 1 Department of Environmental Science, College of Environmental Science and Engineering, Donghua University, Shanghai 201620, China; cpeng@dhu.edu.cn (C.P.); shencs@dhu.edu.cn (C.S.); 2 Key Laboratory of Water Pollution Control and Environmental Safety of Zhejiang Province, Zhejiang University, Hangzhou 310058, China; 3 State Environmental Protection Engineering Center for Pollution Treatment and Control in Textile Industry, Shanghai 201620, China; 4 Department of Environmental Engineering, College of Environmental and Resource Sciences, Zhejiang University, Hangzhou 310058, China; 21614083@zju.edu.cn (S.Z.); 21614035@zju.edu.cn (W.Y.); hhzju@zju.edu.cn (H.H.)

**Keywords:** metal-based nanoparticles, natural organic matter, aggregation, sedimentation, dissolution, speciation

## Abstract

Many studies have shown the effect of solution chemistry on the environmental behavior of metal-based nanoparticles (NPs), except CuO NPs. Here, we investigated the agglomeration, sedimentation, dissolution, and speciation of CuO NPs by varying pH, ionic strength, ionic valence, and natural organic matter (NOM). The results showed that as the pH moved away from 6, the size of CuO agglomerates decreased, along with the enhanced NP suspension stabilization, due to the increase of electrostatic repulsive force. Increasing ionic strength and valence intensified the agglomeration and sedimentation of CuO NPs because of the compression of electrical double layers. The presence of humic acid and citric acid enhanced the dispersion and stabilization of CuO NP suspension, but l-cysteine showed a different impact. Decreasing pH, increasing ionic strength and all NOM improved the dissolution of CuO NPs, but the divalent electrolyte (CaCl_2_) inhibited the Cu^2+^ release from CuO NPs compared to the monovalent electrolyte (NaCl). In addition, X-ray absorption near edge structure (XANES) analysis demonstrated that the presence of l-cysteine transformed more than 30% of CuO NPs to Cu(I)-cysteine by coordinating with thiol group. This study can give us an in-depth understanding on the environmental behavior and fate of CuO NPs in the aquatic environment.

## 1. Introduction

Metal-based nanoparticles (MNPs), as an important category of engineered nanomaterials, have had a 30% market share of the consumer product nanotechnology, as early as 2009 [1]. As one of the most important MNPs, copper oxide nanoparticles (CuO NPs) are extensively used in energy storage, sensors, surfactants, catalysts, and antimicrobial agent in various industries, agricultural activities and environmental remediation, due to their specific electrical, thermal, catalytic, and antibacterial properties [2,3,4]. The annual global production of Cu-based NPs was estimated to be 570 tons/year in 2014 and would be 1600 tons by the year of 2025 [5]. However, the widespread application of CuO NPs is leading to the release of NPs from point and non-point sources into the environment, especially the aquatic environment. The rising environmental concentration of NPs also increases the chances of exposure to NP pollution for organisms [6]. Previous studies have demonstrated that CuO NPs can attach on the surface of organisms and penetrate into the body of organisms, meanwhile inducing cell membrane damage, mitochondrial injury, DNA damage, and even cell death [7,8,9,10]. However, the persistence, bioavailability/bioabsorption, reactivity, and toxicity of NPs are largely determined by the environmental behavior of NPs. Thus, it is of significance to understand the transformation of CuO NPs in the aquatic environment for evaluating the environmental and ecological risks of CuO NPs.

It is still disputable that the soluble Cu or the nano effect should be attributed to the toxicity of CuO NPs to organisms. For instance, the dissolution of CuO NPs might be the primary reason causing the toxicity of CuO NPs to *Pseudokirchneriella subcapitata* [11]. Both the impaired growth of *Triticum aestivum* and the oxidative stress were induced by the dissolved Cu^2+^ from CuO NPs [12]. A recent study also pointed out that the toxicity of CuO and soluble Cu were similar in culture medium and lymphocyte cells, while the nano-specific effect of CuO NPs could be negligible [13]. Yet, Lee pointed out that the average genetic identity of *Fagopyrum esculentum* exposed to CuO NPs was not caused by the toxicity of Cu^2+^ released from CuO NPs [14]. The modulation of antioxidant enzymes in rice seedlings treated with CuO NPs suggested that the defense mechanism was activated to prevent oxidative stress from CuO NPs [15]. Similarly, we found that the adverse effects of CuO NPs on rice seedlings mainly resulted from the generation of reactive oxygen species (ROS) and oxidative stress induced by CuO NPs [16]. In fact, the different nanotoxicities are largely attributed to the widely varied environmental parameters of exposure solution. The solution chemistry plays a critical role in modulating the nanotoxicity and the interfacial interactions between NPs and organisms. Many studies have shown that pH, ionic strength, and ionic valence affected the aggregation, sedimentation, and dissolution of MNPs such as ZnO NPs, Ag NPs, TiO_2_ NPs, and CeO_2_ NPs [17,18,19,20], but the effect of solution chemistry on the transformation of CuO NPs, and its underlying mechanism, is not fully understood.

MNPs can inevitably interact with natural organic matter (NOM) in the natural water body and soil [21]. NOM adsorbing on the NP surface may change the physicochemical properties of NPs and their interfacial tension or action energy, which consequently alters the properties and behavior of MNPs to a large extent. NOM contains various functional groups, such as mercapto, phenols, quinones, aldehydes, ketones, and carboxyl groups, making their interactions with NPs very complex [22]. Humic acid, as an ubiquitous NOM, has been widely investigated in its interaction with MNPs. For example, low concentration of Suwannee River humic acid (SRHA) accelerated the agglomeration and sedimentation of ZnO NPs, while high SRHA reversed the charge of ZnO NPs and enhanced their stability [23]. Moreover, NOM with small molecule weights, such as citric acid, which may be secreted by aquatic plants, significantly enhanced the dissolution of ZnO NPs in the aquatic environment [24]. Additionally, the sulfhydryl-containing organic compounds (thiols), such as cysteine in the natural water, also altered the aggregation and dissolution of Ag NPs [25]. Yet, the knowledge on how NOM with high or low molecule weights influence the transformation of CuO NPs is still limited.

In this study, we investigated the agglomeration, sedimentation, dissolution, and speciation of CuO NPs by varying environmental parameters including pH, ionic strength, ionic valence, and NOM. Commercially bare CuO NPs were chosen due to their wide applications. Three types of NOM (humic acid, citric acid, and l-cysteine) were selected owing to their universal existence in the natural water and soil, and their specific functional group. Dynamic light scattering (DLS) measurement was performed to characterize the size distribution of CuO NPs. The Derjaguin–Landau–Verwey–Overbeak (DLVO) theory was used to analyze the interaction energy between CuO NPs for explaining the aggregation mechanism of NPs. The influence of pH, monovalent (NaCl), and divalent (CaCl_2_) electrolytes, and NOM on the colloidal stability and dissolution of CuO NPs were studied. In addition, X-ray absorption near edge structure (XANES) analysis was applied to determine the chemical form of CuO NPs in the presence of NOM. Overall, this work examines the effects of solution chemistry on the four primary aqueous processes of CuO NPs and reveals their underlying mechanisms, which can help us to gain an in-depth understanding of how CuO NPs may behave in the aquatic environment.

## 2. Results

### 2.1. Properties of CuO NPs

Figure S1 shows the micrograph of CuO NPs by transmission electron microscopy (TEM). The primary diameters of 100 particles in TEM micrographs were randomly selected to measure the size distribution of CuO NPs. CuO NPs have a spherical or elliptical shape with an average particle size of 42.9 ± 21.1 nm. The hydraulic diameter and zeta potential of CuO NPs in Milli-Q water were 240 ± 23 nm and −18.33 ± 2.70 mV, respectively. The absorbance peak for CuO NP suspension was at 240 nm (Figure S2A).

### 2.2. Effect of pH on the Agglomeration of CuO NPs

**Figure 1:**
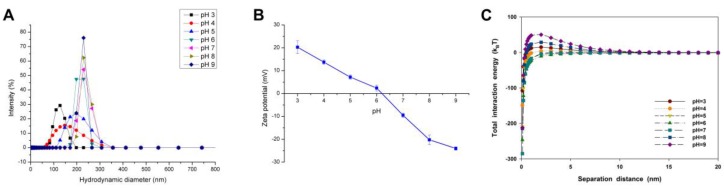
Size distribution (**A**); zeta potential (**B**); and calculated Derjaguin–Landau–Verwey–Overbeak (DLVO) total interaction energy (**C**) of CuO nanoparticles (NPs) (100 mg/L) in the aqueous solution with different pH conditions and a constant ionic strength of 10 mM NaCl. The values of zeta potential were given as mean ± SD of triplicate samples.

The initial particle size and average hydraulic diameter of CuO NPs increased with higher pH in the solution with 10 mM NaCl (Figure 1A). For instance, the hydraulic diameter of CuO NPs was only 124.68 ± 1.18 nm at pH 3, but about 270 nm in the alkaline condition. Moreover, varying pH directly changed the surface charge and density of CuO NPs (Figure 1B). The pH of zero point of charge (pH_zpc_) of CuO NPs was 6.21, which means a poor stability of CuO suspension near pH 6. As the pH moved away from pH_zpc_, the particle size of NP agglomerates was decreased along with the enhanced NP suspension stability, especially when pH was lower than 5. 

The DLVO theory was used to calculate the interaction energy for CuO NPs at different pH. The van der Waals (vdW) force of CuO NPs was gradually enhanced with increasing solution pH (Figure S3A). Yet, various pH values had differing impacts on the electrostatic repulsive force. The electrostatic repulsive force between NPs was decreased with the decline of pH under alkaline conditions, but increased with lower pH in the acidic solution (Figure S3B). The total interaction energy between CuO NPs under varying pH showed a similar trend with the electrostatic repulsive force, thus, the NP aggregation was mainly controlled by the electrostatic repulsive force (Figure 1C). There was a big energy barrier up to 50 k_B_T between CuO particles at pH 9, while almost no energy barrier could be found between particles at pH 5, 6, and 7.

### 2.3. Effect of Ionic Strength and Ionic Valence on the Agglomeration of CuO NPs

**Figure 2:**
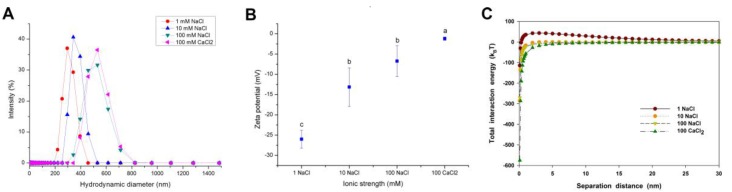
Size distribution (**A**); zeta potential (**B**); and calculated DLVO total interaction energy (**C**) of CuO NPs (100 mg/L) in the aqueous solution with different ionic strength (1, 10, and 100 mM NaCl) and ionic valance (Na^+^ and Ca^2+^) at a neutral pH. The values of zeta potential were given as mean ± SD of triplicate samples. Different letters in Figure 2B indicate significant differences among the treatment means (*p* < 0.05).

Figure 2A,B present the size distribution and zeta potential of CuO NPs measured over a range of NaCl and CaCl_2_ concentrations. The hydraulic diameter of CuO NPs was increased with elevating ionic strength, indicating that high ionic strength enhanced the agglomeration of NPs. At 100 mM NaCl, the average hydraulic diameter of CuO NPs was even as high as 508 nm (Figure 2A). CuO NPs were negatively charged over the whole range of NaCl and CaCl_2_ concentrations (Figure 2B). However, the zeta potential of CuO NPs gradually climbed to zero potential with increasing NaCl concentrations. Compared with the same concentration of monovalent electrolyte, the presence of divalent electrolyte (CaCl_2_) showed a significant positive effect on the increase of NP hydraulic diameter and zeta potential (Figure 2B), which indicates that the divalent electrolyte further reduced the stability of NP suspension and promoted the aggregation of NPs.

The interaction between CuO NPs was quite repulsive at 1 mM NaCl (Figure 2C), while the vdW energy of CuO NPs was enhanced, and the repulsive electrical double layer (EDL) energy of CuO NPs was decreased with the increasing ionic strength, causing a net zero interaction force above 10 mM NaCl (Figure S4). At 100 mM CaCl_2_, the vdW interaction energy of NPs was dramatically increased, but the electrostatic interaction energy was decreased to zero in the comparison with NaCl (Figures S4 and 2C). Thus, the total interaction energy between NPs was dominated by the vdW force. The result indicates that both high ionic strength and high ionic valence led to the compression of the electrical double layers (EDLs), and finally reduced the electrostatic repulsive force and enhanced the vdW force, which caused the decrease of the total energy barrier between CuO particles.

### 2.4. Effect of NOM on the Agglomeration of CuO NPs

**Figure 3:**
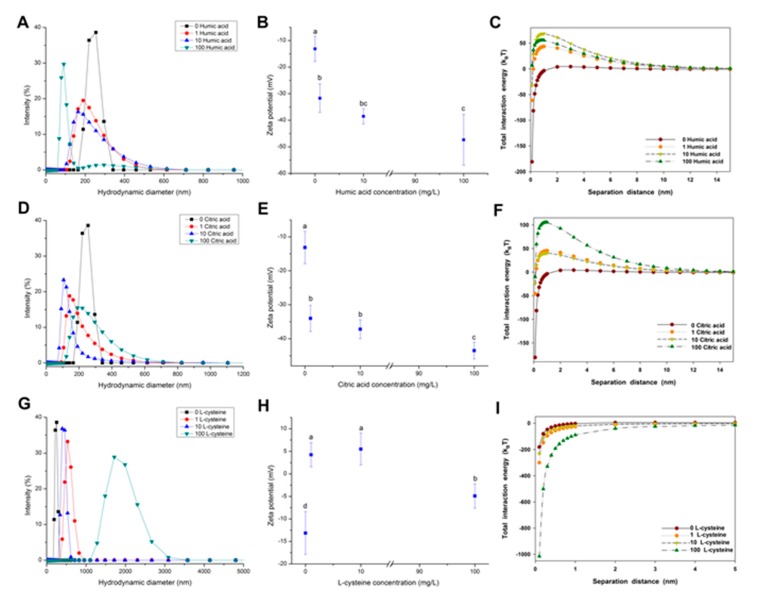
Size distribution (**A**,**D**,**G**); zeta potential (**B**,**E**,**H**); and calculated DLVO total interaction energy (**C**,**F**,**I**) of CuO NPs (100 mg/L) in the aqueous solution with 1, 10, and 100 mg/L humic acid (**A**–**C**); citric acid (**D**–**F**) and l-cysteine (**G**–**I**) at a neutral pH with a constant ionic strength of 10 mM NaCl. The values of zeta potential were given as mean ± SD of triplicate samples. Different letters in Figure 3B,E,H indicate significant differences among the treatment means (*p* < 0.05).

The effect of NOM on the aggregation of CuO NPs was determined by size distribution, zeta potential, and total interaction energy, as shown in Figure 3. A size decrease was observed after the addition of humic acid (Figure 3A), indicating that the presence of humic acid alleviated the agglomeration of CuO NPs. For example, the presence of 100 mg/L humic acid decreased the average hydraulic diameter of CuO NPs from 240 nm to 110 nm. The addition of humic acid also drastically reduced the zeta potential of CuO NPs from −13 mV to −47 mV in a dose-dependent relationship (Figure 3B). The increasing negative charges indicate more humic acid adsorbed on the surface of CuO NPs. CuO NPs had a net repulsive energy barrier approaching zero in the absence of NOM (Figure 3C). The addition of humic acid induced a dose-dependent decline in the vdW energy and an increase in the repulsive EDL energy of CuO particles (Figure S5). The repulsive EDL energy between CuO NPs reached the maximum when the concentration of humic acid was 10 mg/L, rather than 100 mg/L (Figure 3B). Accordingly, 10 mg/L of humic acid maximized the total energy barrier between CuO NPs (Figure 3C). The result shows that the electrostatic repulsive force plays a critical role in the alleviated aggregation of NPs.

The presence of citric acid also reduced the size of CuO NPs. The hydraulic diameter of CuO NPs was reduced to 140 nm at 10 mg/L of citric acid (Figure 3D). Citric acid showed a similar effect on the zeta potential of CuO NPs with humic acid (Figure 3E), suggesting that the stability of CuO NP suspension was improved by citric acid at a neutral pH. The interaction energy curves for CuO NPs as a function of citric acid concentration are shown in Figure 3F. The addition of citric acid resulted in a decrease in the vdW energy between NPs and an increase in the repulsive EDL energy (Figure S6). High citric acid significantly increased the total interaction energy between the particles to over 100 k_B_T (Figure 3F). Hence, the total energy barrier between CuO particles was enhanced with the further addition of citric acid due to the net repulsive energy barrier.

However, the aggregation behavior of CuO NPs in the presence of l-cysteine was dissimilar from that with humic acid and citric acid, as shown in Figure 3G–I. The presence of l-cysteine in the CuO NP suspension caused a sharp increase of CuO particle size. The size distribution of CuO particles ranged from 324 to 825 nm at low concentrations of l-cysteine, while the hydraulic diameter of CuO particles was even up to 3 μm at 100 mg/L of l-cysteine (Figure 3G). Obviously, the presence of l-cysteine greatly promoted the agglomeration of CuO NPs. The zeta potential measurement showed that low amounts of l-cysteine reversed the surface charge of CuO NPs from negative to positive, then CuO NPs became negatively charged again with further addition of l-cysteine (Figure 3H). Notably, the zeta potential of CuO NPs was in the range of zero potential ±5 mV, suggesting a quite poor stability of CuO suspension with l-cysteine. DLVO theory calculations reveal that the presence of l-cysteine not only enhanced the vdW force of CuO NPs, but also reduced the electrostatic repulsive force (Figure S7). The negative total interaction energy between NPs indicates that the addition of l-cysteine intensified the aggregation of CuO NPs, which is consistent with the change of NP zeta potential.

### 2.5. Sedimentation of CuO NPs Exposed to Varying pH, Electrolyte, and NOM

**Figure 4:**
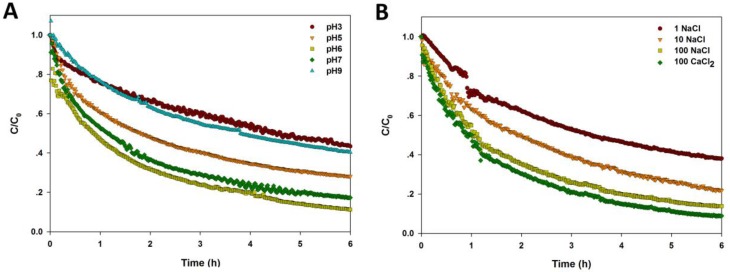

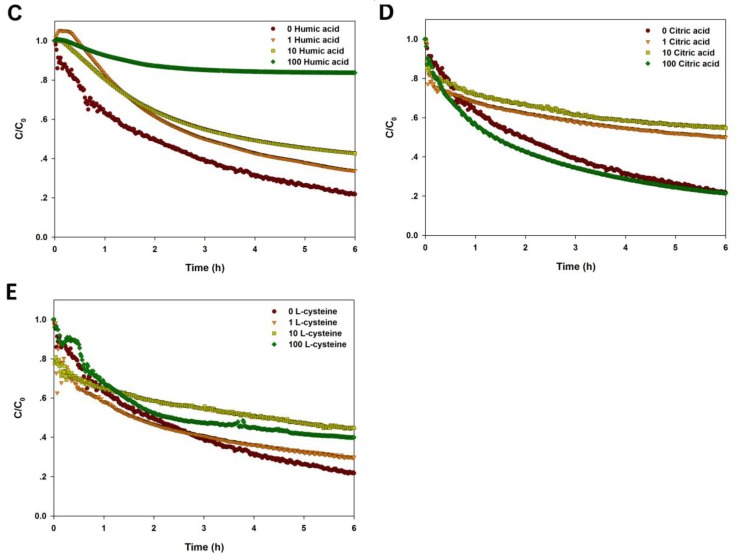
Sedimentation of CuO NPs (100 mg/L) in the solution with varying pH (**A**); ionic strength and ionic valence (**B**); and different amount of humic acid (**C**); citric acid (**D**); and l-cysteine (**E**).

The sedimentation curves of CuO NPs under differing pH, electrolyte, and NOM are shown in Figure 4. A steepest descend could be found in the sedimentation curve of NPs at pH 6, near to pH_zpc_, while the NP sedimentation curve slopes gently at pH 3 and 9 (Figure 4A). Also, the settling rate of NPs was highest at pH 6, and the sedimentation rate decreased significantly when the solution pH was far from pH_zpc_ (Table 1). Figure 4B shows that the sedimentation of CuO NPs was enhanced with higher ionic strength. Moreover, the presence of divalent cations (Ca^2+^) further accelerated the NP sedimentation at the same ionic strength, indicating that the higher charges of cations lead to the worsening stability of CuO NPs. The sedimentation rate, *k*, also increased sharply with the increase of ionic strength and ionic valence (Table 1).

As shown in Figure 4C and Table 1, the presence of humic acid significantly reduced the sedimentation rate of CuO NPs with a dose-dependent response. When the concentration of humic acid was up to 100 mg/L, the stability of CuO NPs was enhanced markedly, and less than 80% of CuO NPs was precipitated after 6 h, whereas, the first-order kinetics model did not fit well with the sedimentation curve. Lower concentrations of citric acid (1 and 10 mg/L) significantly reduced the sedimentation of CuO NPs in solution compared to the control, but high citric acid (100 mg/L) had little effect on the settling performance of NPs in water (Figure 4D). The fitting result shows that the sedimentation rate of NPs was decreased dramatically with increasing citric acid, but the effect was weakened with high citric acid (Table 1). The sedimentation curve of CuO NPs became steeper within 30 min in the presence of l-cysteine (1 and 10 mg/L), suggesting that lower amount of l-cysteine accelerated the NP sedimentation in a short time (Figure 4E). Yet, the stability of NPs was still promoted by l-cysteine over time. Also, the sedimentation rate of CuO NPs was increased by the presence of l-cysteine (Table 1).

### 2.6. Dissolution of CuO NPs Exposed to Varying pH, Electrolytes, and NOM

**Figure 5:**
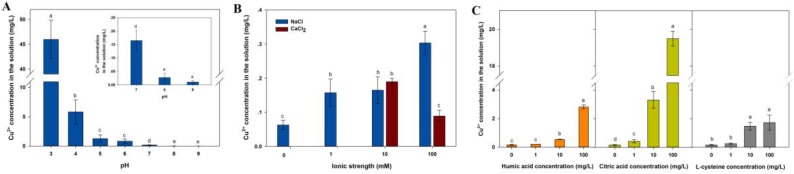
Dissolution of CuO NPs (100 mg/L) in the solution with varying pH (**A**); ionic strength, and ionic valence (**B**); and different amount of humic acid, citric acid, and l-cysteine (**C**). The insert in Figure 5A was the enlarged figure from pH 7 to 9. The values of Cu concentration were given as mean ± SD of triplicate samples. Different letters indicate significant differences among the treatment means (*p* < 0.05).

In our batch experiments, the soluble Cu released from CuO NPs (100 mg/L) was up to 56.25% of the total Cu at pH 3, but only 0.19% at pH 7, showing a negative correlation between solution pH and CuO NP solubility (Figure 5A). NaCl significantly promoted the dissolution of CuO NPs (Figure 5B). The Cu^2+^ released from CuO NPs in 100 mM NaCl solution was five times more than that in control. Whereas, compared to NaCl at the same ionic strength, CaCl_2_ with a higher ionic valence showed no significant effect on the dissolution of CuO NPs at low ionic strength (10 mM), but slightly inhibited the release of Cu^2+^ at high ionic strength (100 mM). The result suggests that not only the ionic strength, but also the type of electrolyte impacted the release of CuO NPs. The amount of dissolved Cu released from CuO NPs was markedly promoted by the presence of the three NOM types, which was also positively correlated with NOM contents (Figure 5C). In the absence of NOM, the nominally dissolved Cu concentration in the CuO NP suspension was only 0.16 mg/L. While in the presence of 100 mg/L NOM, the concentration of Cu was increased to 2.80 ± 0.14 mg/L for humic acid, 19.48 ± 0.41 mg/L for citric acid, and 1.73 ± 0.52 mg/L for l-cysteine, suggesting a strong effect of NOM, especially citric acid, on promoting the soluble Cu released from CuO NPs. Thus, pH and NOM are critical factors in the dissolution of CuO NPs with more soluble Cu occurring at lower pH and higher NOM.

### 2.7. Speciation of CuO NPs in the Presence with NOM

**Figure 6:**
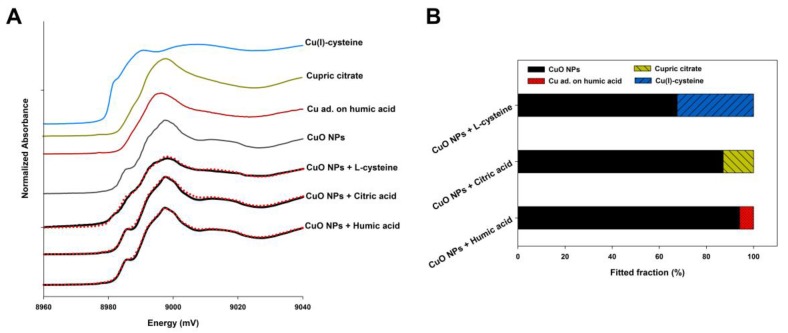
The X-ray absorption near edge structure (XANES) Cu K-edge spectra of model compounds and CuO NPs in the solution with NOM (**A**); the red dashed lines are the linear fitting results. Cu ad. on humic acid: Cu adsorbed on the humic acid. Results of fitting the Cu K-edge XANES spectra of CuO NP samples exposed to NOM using a linear combination of the data for the model compounds (**B**).

Figure 6 shows the oxidation state and speciation of Cu element and their proportions in the CuO NP samples with NOM. The XANES analysis showed that nearly 95% of Cu in the particles was still in the form of CuO, while a small amount of Cu element was combined with humic acid, which means a weak effect of humic acid on the chemical transformation of CuO NPs. The presence of citric acid in a neutral environment resulted in that 12.9% of total Cu from CuO NPs was transformed to cupric citrate, but most Cu element remained in their original speciation. However, CuO NPs that were exposed to l-cysteine exhibited a quite higher percentage of transformed Cu than that of humic acid and citric acid. After the addition of l-cysteine for 48 h, up to 32.4% of Cu in CuO NPs was reduced to Cu(+I) and coordinated with thiol groups of l-cysteine forming Cu(I)-cysteine. All R-factors for the linear combination fitting (LCF) analysis were less than 0.0014, manifesting a good fit of the data.

## 3. Discussion

The NP agglomeration in water not only changes the distribution of NPs in the environment, but also affects the migration characteristics of NPs and their interactions with organisms. To a large extent, the aggregation of NPs depends on pH, ionic strength, and electrolyte type, because they largely determine the surface charge and charge density of NPs. Most MNPs have surface functional groups such as oxides and hydroxide groups, which can combine with hydrogen ions or hydroxyl groups in the aquatic environment. Thus, the change of solution pH may cause the surface charge reversal of NPs, further strongly affecting the agglomeration of NPs. Hence, the aggregation and sedimentation of CuO NPs in solution were increased via reducing the total energy barrier between CuO NPs when the solution pH was close to pH_zpc_ of CuO NPs (Figure 1 and Figure 4A). Whereas, the big energy barrier of CuO particles at pH 9 contradicts their big hydraulic diameters. This can be explained by the fact that the interaction energy between CuO NPs, which mainly is the electrostatic repulsive force, was controlled by the surface charge density of CuO NPs. In fact, the hydraulic diameter of CuO NPs was mainly controlled by the solution pH, especially under acidic conditions.

Enhancing ion strength and valence led to the increase of vdW force and the decrease of electrostatic repulsion between CuO NPs, which reduced the energy barrier between NPs, promoted the agglomeration of CuO NPs, and accelerated the settling of NPs (Figure 2 and Figure 4B). In the case of ionic solute effects, increasing ionic concentration weakens the repulsion force between double electron layers, which decreases the energy barrier between the particles and further intensifies agglomeration. Electrostatic disturbances are strongly influenced by the ionic valence [26]. The increased Debye length caused by the enhancing valence reduces the electrostatic repulsive force and further promotes the NP agglomeration [20]. When the ion concentration is close to the critical coagulation concentration (CCC), the exclusion of the energy barrier may completely disappear, resulting in rapid agglomeration of particles. It was reported that alginate-coated hematite NPs were agglomerated by electrostatic disturbances of NaCl and CaCl_2_ in the solution [27]. French et al. found that the agglomeration rate of TiO_2_ NPs in the solution with divalent cations (Ca^2+^) was faster than that in the solution containing monovalent cations (Na^+^) with the same pH and ionic strength [20]. 

The interaction mechanism of NOM and MNPs mainly involves electrostatic interaction, ligand exchange, hydrophobic interaction (such as vdW force), hydrogen bonding, and cation bridges [22]. In the molecular structure of humic acid, the nucleus contains aromatic rings, and heterocyclic and polycyclic compounds, while the edge consists of abundant carboxyl, carbonyl, phenolic hydroxyl, amine groups, and other active groups. Under low ionic strength, same surface charges of humic acid and CuO NPs cause the decrease of vdW force, but the increase of repulsive electrostatic forces between CuO NPs. Therefore, it is the electrostatic interaction that resulted in the weakened agglomeration, enhanced stability, and decreased settling velocity of CuO NPs. Previous studies have shown that the formation of humic acid and Fe NP complexes involves electrostatic interactions [28]. Similarly, fulvic acid, with abundant negatively-charged carboxyl groups, was strongly adsorbed on borate-coated Ag NPs by electrostatic attraction, thereby alleviating the agglomeration of colloidal particles [29]. We also found that increasing amount of humic acid improved the stability of CuO NPs and reduced the sedimentation rate of NPs. However, the energy barrier between CuO NPs did not reach the maximum when the humic acid concentration was up to 100 mg/L (Figure 3C), indicating that the electrostatic interaction between NPs cannot fully explain the interaction mechanism of humic acid and CuO NPs. Notably, humic acid can also affect the stabilization of CuO NP suspension via steric hindrance. For instance, when the ionic strength was below the CCC, the steric hindrance caused by the addition of humic acid inhibited the agglomeration of NPs [30]. The agglomeration of ZnO NPs will still be reduced in the presence of humic acid, even with a high ionic strength, due to NOM coating on the NP surface [21]. Hence, both electrostatic interaction and steric hindrance of humic acid inhibit the agglomeration and sedimentation of CuO NPs.

Citric acid, as a low molecular weight tricarboxylic acid compound, is mostly deprotonated, generating free citrate after three stages of the ionization in a neutral pH environment, because the pK_a_ values of citric acid are 3.13, 4.72, and 6.33 [16]. A part of carboxylic groups in the citric acid can replace the abundant hydroxyls on the metal oxide surface and combine with metal cations, forming a single molecular adsorbed layer. Then, the particle surface is negatively charged, thus promoting the dispersion of CuO NPs in solution via the electrostatic repulsion [31]. Arancon et al. reported that the average particle size of Au NPs decreased from 84 nm to 76 nm after the addition of citric acid [32]. It can also be found that the electrostatic repulsive force between CuO NPs increased sharply in the presence of citric acid, which significantly inhibited the NP agglomeration and improves the NP stability (Figure 3D,E). This indicates the role of electrostatic interaction in citric acid maintaining the stabilization of CuO NP suspension. Nonetheless, a high citric acid concentration (100 mg/L) had little impact on the sedimentation of CuO NPs compared to the control. The reason is that once they have contact with citric acid on a long term, CuO NPs could be dissolved to Cu^2+^, which may combine with citrate, forming copper citrate (Figure 6). On one hand, the particle size of dissolved CuO NPs will decrease to a certain extent. On the other hand, the amount of copper citrate may be partially attached to the NP surface, which leads to the increase of particle size. Both these two reactions can affect the sedimentation of CuO NPs in water, but it is difficult to quantify the two processes, since they may interact or proceed at the same time.

l-Cysteine, as a common amino acid in organisms, has a strong binding affinity to metal ions such as Cu^2+^, Ag^+^, and Hg^+^, producing an insoluble thiolate complex, due to its unique thiols (–SH). Cysteine can be adsorbed on the surface of Ag NPs and Au NPs by the formation of Ag (I)–S and Au–S bonds, which cause the agglomeration of NPs via electrostatic interactions between cysteine-bound NPs [25,33]. Additionally, cysteine-promoting NP agglomeration may be induced by the hydrogen bonds of carboxyl groups in cysteine adsorbed on adjacent particles [34]. However, Sudeep et al. and Zhang et al. argued that the interaction of zwitterions was the primary agglomeration mechanism [35,36]. Vallée et al. also found the presence of neutral and zwitterions during the adsorption of glutathione containing cysteine on Au [37]. Thus, cysteine may promote the agglomeration of CuO NPs by the hydrogen bonding between particles and the electrostatic interaction between zwitterions (Figure 3G–I). Furthermore, the released Cu^2+^ from CuO NPs can catalyze the oxidation of cysteine, inducing the rapid formation of cysteine disulfide [38]. In fact, the cysteine-bound Cu complex forms a cyclic structure with the –SH bond of cysteine to act as a bridging ligand once Cu (II) binds to the thiol ligand of cysteine [39]. Since Cu^2+^ on the NP surface may also participate in the reaction, the complex formation may be coated on the surface of CuO NPs, which increases the hydraulic diameter of NPs and promotes the NP agglomeration. The results of the zeta potential measurement also showed that the surface charge of NPs was close to zero with the cysteine addition (Figure 3H), and the suspension stability declined drastically (Figure 4E). Thus, thiol groups in the cysteine are prone to combine with Cu in CuO NPs to perform a strong coordination, rather than the electrostatic interaction between NPs (Figure 6). Whereas, the presence of cysteine alleviates the sedimentation of NPs to a certain extent. It has also been found that the lysine consisting of cysteine modified the surface of Au NPs, and further improved the stability of quantum dots (QDs) [40]. 

Dissolution, as a common character of most MNPs, plays a key role in the fate and toxicity of MNPs in the environment. The toxic effects of MNPs on organisms partly result from their released metal ions [41]. Actually, the dissolution depends on the solubility of material in the solvent, and the concentration difference between solute surface and background solution [42]. The dissolution kinetics of MNPs are directly affected by pH (Figure 5A). Under strong acid condition, CuO NPs can react with high concentrations of H^+^ in the solution and release a large amount of Cu^2+^, while under weakly acidic conditions, only a small amount of H^+^ forms contacts with CuO NPs, generating Cu(OH)^+^ , as described in our previous study [43]. 

The addition of electrolyte changes the dissolution of MNPs by possible chemical reactions and its influence on the agglomeration. Previous studies have shown that the destabilization caused by the addition of electrolyte induced Ag NPs to dissolve immediately [44,45]. We also found that the increasing ionic strength contributes to the dissolution of CuO NPs in water (Figure 5B), because the salt effect causes a higher solubility of metal oxides in the electrolyte solution than that in pure water [46]. Yet, Gunawan et al. found no significant effect on the solubility of CuO NPs before and after adding NaCl to deionized water [47]. In addition, the dissolution of MNPs is largely dependent on the type and concentration of electrolyte. The results showed that the effect of cation ions Na^+^ and Ca^2+^ on the dissolution of CuO NPs was not significantly different at low ionic strength, however, divalent cations (Ca^2+^) had a lesser impact on the CuO NP dissolution when compared to monovalent cations (Na ^+^) at high ionic strength. The reason may be that the surface charge of CuO NPs is almost completely neutralized by Ca^2+^ to form a more stable agglomerate, which decreases the specific surface area of particles and accelerates their sedimentation, and further leads to a decline in the possibility of partial CuO contacting with H^+^ in water.

The presence of humic acid promoted the dissolution of CuO NPs with a dose-dependent effect (Figure 5C). It was reported that the sensitization of humic acid is beneficial to the release of Cd and Se by quantum dots (QDs) in solution with low concentration of humic acid. Moreover, some functional groups from humic acid, such as phenolic and carboxylic groups, can complex with metal ions [48]. Once CuO NPs begin to release Cu^2+^, more crystal planes and surface defects may be exposed to humic acid, which further facilitates the Cu^2+^ release from CuO NPs [48]. Similarly, both the presence of humic acid and its increasing amount improved the Zn^2+^ dissolution level of ZnO NPs despite pH and synthesis methods [48,49]. Dissolved NOM, such as Suwannee River fulvic acid (SRFA), promotes the dissolution of CuO NPs in water [50]. We also found that citric acid significantly contributes to the release of CuO NPs, even at neutral pH (Figure 5B). This can be explained by the fact that citric acid can constantly release H^+^ via first, second, and third stage ionizations in water, driving the dissolution of CuO NPs. Furthermore, free citrate as a complex ligand can interact with NPs, including polarization and weakened metal–oxygen binding [24]. l-cysteine in solution promoted the release of Cu^2+^ from CuO NPs. In this reaction, thiol groups in the cysteine can interact with Cu(I) forming polymers via bridging thiolate sulfur [51]. Likewise, cysteine significantly increased the dissolution rate and solubility of ZnO NPs and Ag NPs [25,52]. Additionally, NOM may further promote the dissolution of CuO NPs via the complexation and coordination of Cu^2+^ [52].

## 4. Materials and Methods

### 4.1. Characterization of CuO NPs

CuO NPs have an average particle size of 40 nm, a specific surface area of 131 m^2^/g, and a purity of 99.9% according to the product information from the manufacturer (Beijing Nachen Technology Co., Beijing, China). CuO NP powders were embedded in the resin and heated overnight at 70 °C, then were sliced into 90 nm with a Reichert Ultra Cut E microtome (Leica Microsystems AG, Wetzlar, Germany) and mounted on a nickel grid. The morphology and size of CuO NPs were observed using a TEM (Hitachi, H-7650, Tokyo, Japan) with an operating voltage of 60 kV. 

### 4.2. Solution Chemistry

Milli-Q water with a constant ionic strength of 10 mM NaCl was used as bulk solution in the study of pH and NOM effects on the stability of CuO NPs. The bulk solution pH was adjusted with HCl or NaOH solution. The ionic strength and ionic valence of Milli-Q water were adjusted with NaCl or CaCl_2_ solution at a neutral pH. A stock solution of humic acid (Sigma-Aldrich Co., St Louis, MO, USA) with an initial mass concentration of 1000 mg/L was prepared by dissolving humic acid in Milli-Q water and adjusting solution pH to 7.0 ± 0.1. Then the solution was stirred overnight, and filtered through a 0.45 μm cellulose membrane [53]. The actual dissolved humic acid concentration in the solution was measured at a wavelength of 254 nm using a UV–vis spectrophotometer (Jinghua Technology Instrument Co., Model 752C, Shanghai, China) [48]. The stock solutions of citric acid and l-cysteine (1000 mg/L) were prepared by dissolving citric acid and l-cysteine in Milli-Q water and adjusting solution pH to 7.0 ± 0.1.

### 4.3. Aggregation and Zeta Potential Measurements

A stock solution of CuO NPs (1000 mg/L) was prepared with Milli-Q water (18.2 MΩ; Millipore, Bedford, MA, USA), then was sonicated at 100 W and 40 kHz for 30 min at 25 °C. We added the stock solution of CuO NPs into the bulk solution with different pH, ionic strength and valence, and NOM. All experiments were performed at a concentration of 100 mg/L CuO NPs. The size distribution and zeta potential of CuO NPs were measured using Zetasizer Nano ZS-90 (Malvern Instruments Ltd., Worcestershire, UK) after the mixed CuO suspensions were allowed to equilibrate for 2 h at room temperature. The refractive index and viscosity of CuO NP suspension were set as 1.330 and 0.8872 cP, respectively. The size distribution of NPs was measured over time via DLS. The data processing mode was the multi-mode and high resolution. The zeta potential was measured via the light scattering phase analysis using Zetasizer Nano ZS-90 [54].

### 4.4. Analysis of the Interaction between Particles by DLVO Theory

We used the DLVO theory to analyze the aggregation behavior of CuO NPs under various environmental conditions. Classic DLVO is based on the interaction energy balance that consists of attractive van der Waals (vdW) and repulsive electrostatic forces from the overlap of the electrical double layers (EDL) of interactive surfaces (Equation (1)) [55]. Both vdW attraction and electrostatic repulsive forces are functions of the interaction distance between the two interacting particles, 1 and 2, in water. Besides the hydrodynamic radii, the vdW force is related to the NP Hamaker constant, which is an intrinsic property of NPs. Electrostatic repulsion is related to surface potential of NPs and Debye length, which is a function of ionic strength and electrolytes in water, according to Equation (2):

(1) $$U_{1w2}^{DLVO}=U_{1w2}^{vdW}+U_{1w2}^{EL},$$

(2) $$U_{1w2}^{vdW}(h)=-\frac{A_{H}}{6}\left[\frac{8R_{R}^{2}}{h\left(8R_{R}+h\right)}+\frac{8R_{R}^{2}}{{\left(4R_{R}+h\right)}^{2}}+\ln\frac{h\left(8R_{R}+h\right)}{{\left(4R_{R}+h\right)}^{2}}\right]$$

(3) $$U_{1w2}^{EL}(h)=4\pi \varepsilon \varepsilon_{0}R_{R}\left[\phi_{1}\phi_{2}\exp(-\kappa h)-\frac{1}{4}(\phi_{1}^{2}+\phi_{2}^{2})\exp(-2\kappa h)\right]$$

(4) $$\kappa^{-1}=\sqrt{\frac{\varepsilon \varepsilon_{0}k_{B}T}{2N_{A}Ie^{2}}}$$

where $U_{1w2}^{DLVO}$, $U_{1w2}^{vdW}$ and $U_{1w2}^{EL}$ are the total interaction energy, vdW attractive energy, and electrostatic interaction energy between particles 1 and 2, respectively; *h* is the interacting distance between particles 1 and 2; *A_H_* is the particle 1 to particle 2 Hamaker constant in water (*w*); *R_R_* is the reduced particle radius, *R_R_* = *R*_1_*R*_2_/(*R*_1_ + *R*_2_), *R*_1_ and *R*_2_ are the radius of interacting particles 1 and 2, respectively; *ε* and *ε*_0_ are the dielectric constant of water (78.5, dimensionless) and vacuum (8.854 × 10^−12^ C·V^−1^·m^−1^), respectively; *ϕ*_1_ and *ϕ*_2_ are the normalized dimensionless surface potentials of particles, respectively, and defined as *ϕ_i_* = *zeψ_i_* × *k_B_^−1^T^−1^*, where *ψ*_1_ and *ψ*_2_ are the particle surface potentials; *κ* is the inverse Debye length; *k_B_* is the Boltzmann constant, 1.38 × 10^−23^ J·K^−1^; *T* is the temperature; *N_A_* is the Avogadro’s number, 6.02 × 10^23^ mol^−1^; *I* is the ionic strength in M. *I* = 0.5 × *∑c_i_Z_i_*^2^·∑ciZi2, where *c_i_* ciis the molar concentration of ionic species *i*; *Z_i_* is the valency of *i*th ion; *e* is the unit charge, 1.602 × 10^−19^ C.

### 4.5. Sedimentation Study

A full wave scanning from 200 to 700 nm was carried out to determine the optimum scanning wavelength of CuO NP suspension (100 mg/L) using UV–vis spectrophotometry. The optical absorbance of CuO NP suspension was measured at 240 nm (Figure S1) as a function of time during 6 h with 1 min intervals. All measurements were made at 25 °C in a quartz cuvette with 1 cm light path.

In order to relate the NP sedimentation to the aggregation kinetics of CuO NPs, the sedimentation theory by Stokes was applied to calculate removal rates of NPs from the water phase. Based on the Stokes formula, the settling velocity of spherical particles is proportional to the square of the particle diameter. The first-order kinetics model (Equations (5) and (6)) was obtained by fitting the sedimentation curve, because Figure S8 shows that the first-order model fits the data well (*R*^2^ = 0.9925) [56]:

(5) $$\frac{dC}{dt}=-kC$$

(6) $$\ln C=-kt+\ln C_{0}$$

where *t* is the time; *C*_0_ and *C* are NP concentrations at 0 and *t* timepoints, respectively, which are represented by the optical absorbance at 0 and *t* timepoints; and *k* is the sedimentation rate (h^−1^).

### 4.6. Dissolution Measurements

We prepared CuO NPs (100 mg/L) suspension under varying pH, ionic strength and ionic valence, and NOM content conditions. After 48 h standing at 25 °C, the CuO NP suspension was transferred to an Amicon Ultra-4 10 kDa centrifugal filter tube (maximum pore size ~3 nm, Millipore, Carrigtwohill, Ireland) and centrifuged at 5000× *g* for 40 min. The filtrate was added with 1% HNO_3_, and then the Cu^2+^ concentration was determined by a flame atomic absorption spectrometer (AAS, MKII M6, Thermo Electron, Waltham, MA, USA).

### 4.7. XANES Analysis

CuO NPs associated with NOM were pre-frozen overnight at −70 °C, and lyophilized at −56 °C and 0.280 mbar pressure for 48 h in a freeze-dryer (Alpha1-4LSC, Marin Christ Ltd., Osterode, Germany). CuO–NOM complexes were adhered on 3M tape (Scotch 810, 3M, Saint Paul, MN, USA) and placed on an aluminum sample holder. Cu K-edge XANES spectra of samples and references were collected on the beamline 1W1B at the Beijing Synchrotron Radiation Facility (BSRF, Beijing, China) and the beamline 14W1 at the Shanghai Synchrotron Radiation Facility (SSRF, Shanghai, China). The details of the beamline operating conditions and reference sample preparation have been described in our previous studies [43,54,57]. The spectra of samples were recorded in the transmission mode. The XANES data of samples were processed and analyzed using IFEFFIT Athena software package (Chicago, IL, USA) written by Ravel and Newville.

### 4.8. Statistical Analysis

One-way ANOVA with LSD (least significant difference) test was used to perform the significant analysis (SPSS Version 16.0, SPSS Inc., Chicago, IL, USA). *p* < 0.05 was considered to be significantly different. All data in the figures are the mean ± standard deviation (SD). Each treatment was performed in triplicate.

## 5. Conclusions

Our results demonstrate that the solution chemistry obviously alters the environmental behavior of CuO NPs. Environmental pH determines the surface charge of CuO NPs to a large extent. Agglomeration and sedimentation of CuO NPs were enhanced with increasing ionic strength and valence. Divalent electrolyte (CaCl_2_) slowed down the Cu^2+^ released from CuO NPs compared to monovalent electrolyte (NaCl). Obviously, NOM components and species have different impacts on the NP agglomeration and sedimentation behavior. All of them promoted the dissolution and transformation of CuO NPs in a neutral environment. However, the change of multi-environmental parameters may occur at the same time in a real natural environment, thus, how they simultaneously impact on the fate and environmental behavior of MNPs still needs to be explored in future studies. Additionally, due to the abundance of NOM in the natural environment, the effect of more varieties of NOM on the transformation of MNPs should be considered in the future.

## Figures and Tables

**Table 1:** Dynamic fitting of CuO NPs with different pH, electrolyte, and natural organic matter (NOM) contents in the aqueous solution.

**Environmental Factors**	**pH**					**Ionic Strength (mM)**			
						**NaCl**			**CaCl_2_**
	**3**	**5**	**6**	**7**	**9**	**1**	**10**	**100**	**100**
*k* (*h*^−*1*^)	0.1180	0.2529	0.3054	0.2631	0.1415	0.1975	0.2631	0.4174	0.4260
*R*^2^	0.9840	0.9509	0.9803	0.9932	0.9689	0.9888	0.9932	0.9930	0.9942
**NOM**	**Humic Acid Concentration (mg/L)**			**Citric Acid Concentration (mg/L)**			**l-cysteine Concentration (mg/L)**		
	**1**	**10**	**100**	**1**	**10**	**100**	**1**	**10**	**100**
*k* (*h*^−*1*^)	0.1944	0.1429	0.0266	0.0713	0.0663	0.2188	0.1510	0.0842	0.1288
*R*^2^	0.9621	0.9444	0.7620	0.9209	0.9195	0.9621	0.9364	0.9595	0.8495

## Supplementary Materials

The following are available online at www.mdpi.com/2079-4991/7/10/326/s1. Figure S1: TEM image of CuO NPs, Figure S2: UV-VIS spectra of CuO NPs (100 mg/L) in the solution by a full wave scanning (A); the calibration curve for humic acid quantification using the absorbance measurement at 254 nm (B), Figure S3: Calculated vdW interaction energy (A) and electrostatic interaction energy (B) between two CuO NPs under varying pH condition and a constant ionic strength of 10 mM NaCl, Figure S4: Calculated vdW interaction energy (A) and electrostatic interaction energy (B) between two CuO NPs under varying ionic strength (1, 10, and 100 mM) and ionic valence (Na^+^ and Ca^2+^), Figure S5: Calculated vdW interaction energy (A) and electrostatic interaction energy (B) between two CuO NPs under varying concentrations of humic acid (0, 1, 10, and 100 mg/L), Figure S6: Calculated vdW interaction energy (A) and electrostatic interaction energy (B) between two CuO NPs under varying concentrations of citric acid (0, 1, 10, and 100 mg/L), Figure S7: Calculated vdW interaction energy (A) and electrostatic interaction energy (B) between two CuO NPs under varying concentrations of L-cysteine (0, 1, 10, and 100 mg/L), Figure S8: Fit of the sedimentation data to the Stokes equation.
