# Supplementary material for: Transformation of CuO Nanoparticles in the Aquatic Environment: Influence of pH, Electrolytes and Natural Organic Matter
Source: Nanomaterials (Basel). 2017 Oct 14;7(10):326. doi: 10.3390/nano7100326 (PMC5666491; doi:10.3390/nano7100326)
Supplement: Supplementary file 1 [file nanomaterials-07-00326-s001.pdf]

# Supplementary Materials: Transformation of CuO Nanoparticles in the Aquatic Environment: Influence of pH, Electrolytes and Natural Organic Matter

**Cheng Peng, Chensi Shen, Siyuan Zheng, Weiling Yang, Hang Hu, Jianshe Liu and Jiyan Shi**

## Materials and Methods

*Determination of the absorption wavelength*

The absorption of UV light by CuO NPs within certain concentration range can fit the Lambert-Beer's law, so the sedimentation of CuO NPs can be reflected by the changes in the absorbance. According to the full-wavelength scanning data, the absorption of UV by 100 mg/L CuO NPs reached the maximum at the wavelength of 240 nm (Figure S2A). Thus, all sedimentation experiments were performed at the wavelength of 240 nm. Figure S2B shows that the absorbance of UV by humic acid at 254 nm has a good linear relation with its concentration.

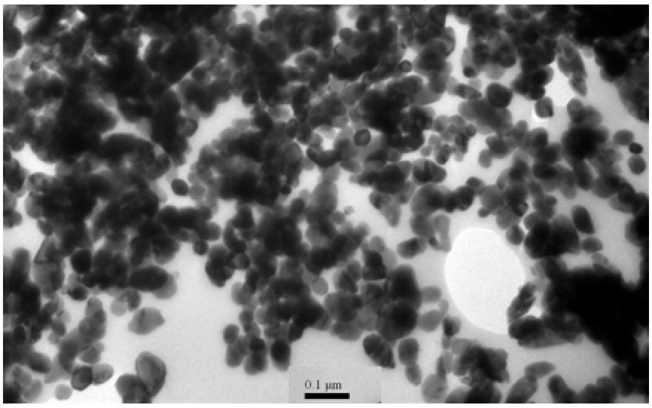


**Figure S1.** TEM image of CuO NPs

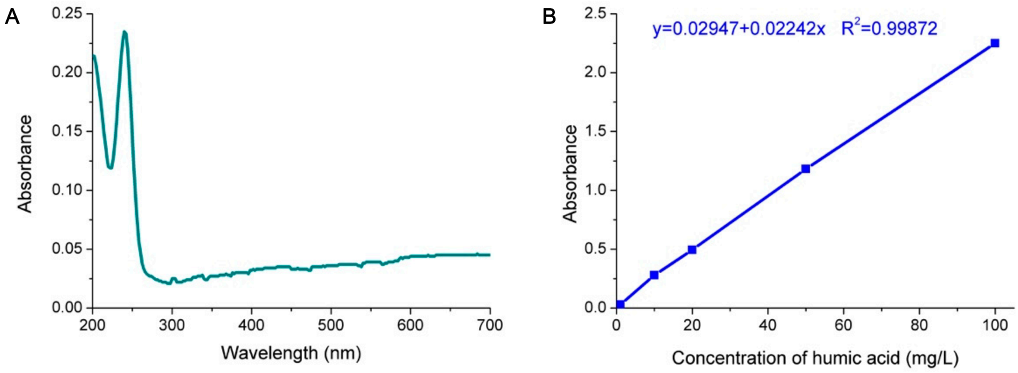


**Figure S2.** UV-VIS spectra of CuO NPs (100 mg/L) in the solution by a full wave scanning (A); the calibration curve for humic acid quantification using the absorbance measurement at 254 nm (B).

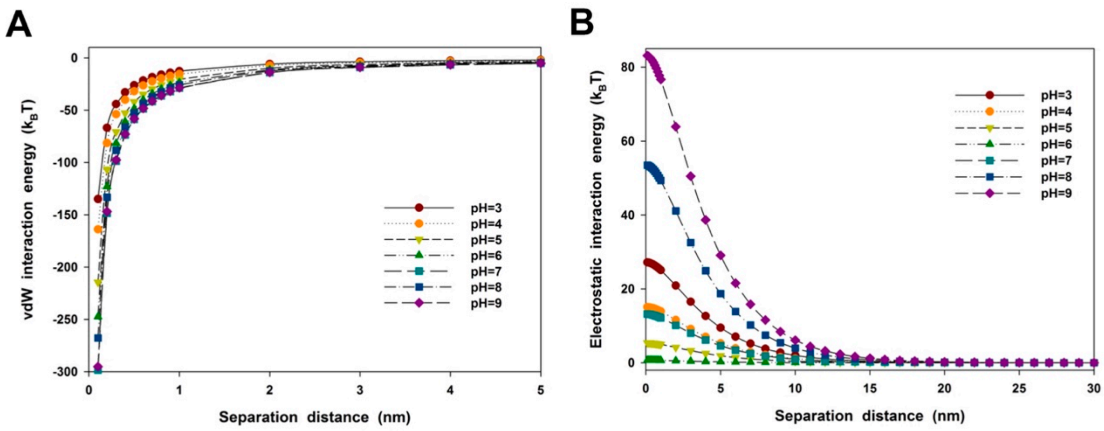


**Figure S3.** Calculated vdW interaction energy (A) and electrostatic interaction energy (B) between two CuO NPs under varying pH condition and a constant ionic strength of 10 mM NaCl.

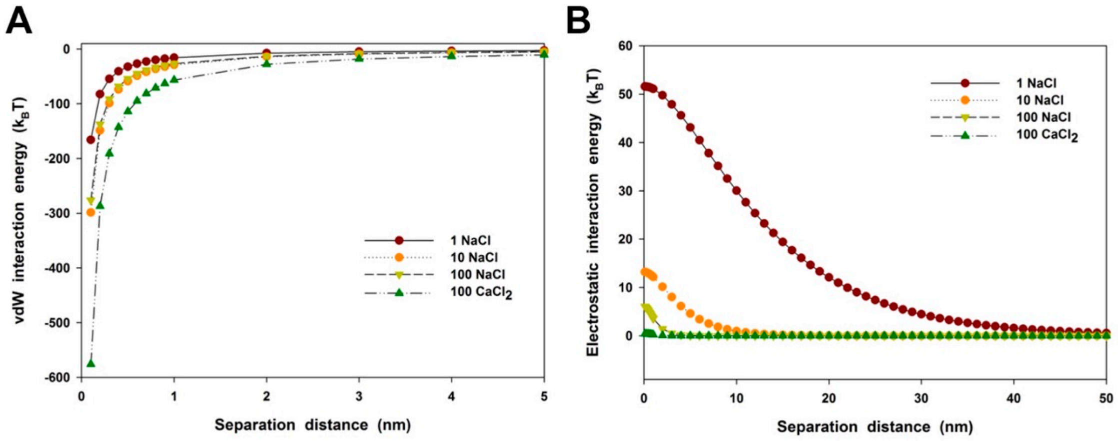


**Figure S4.** Calculated vdW interaction energy (A) and electrostatic interaction energy (B) between two CuO NPs under varying ionic strength (1, 10, and 100 mM) and ionic valence ($Na^+$ and $Ca^{2+}$).

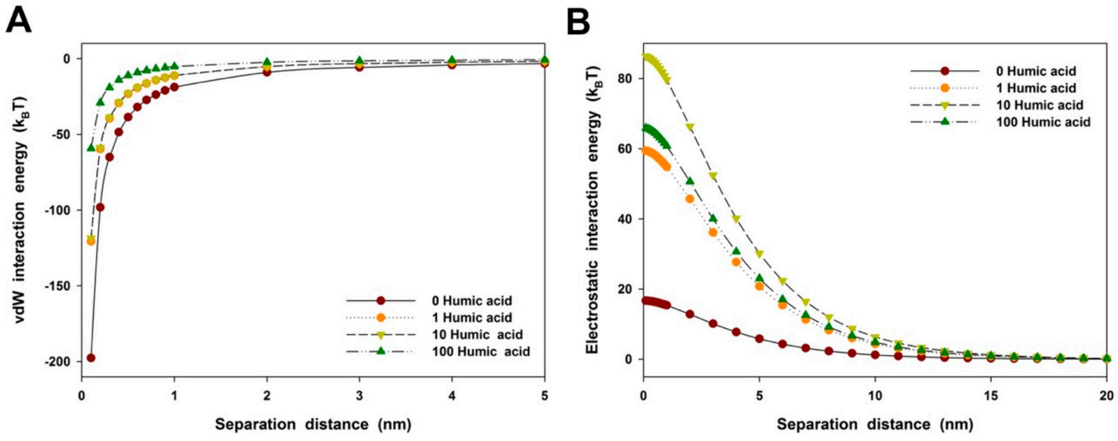


**Figure S5.** Calculated vdW interaction energy (A) and electrostatic interaction energy (B) between two CuO NPs under varying concentrations of humic acid (0, 1, 10, and 100 mg/L).

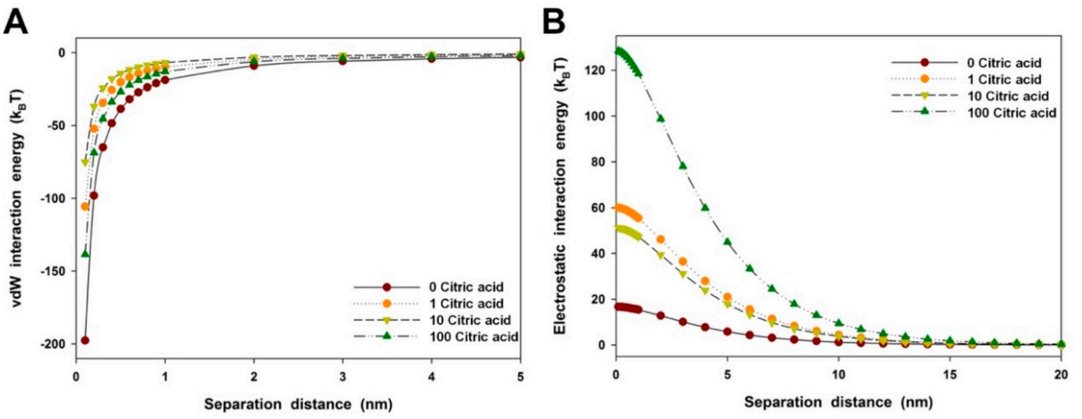


**Figure S6.** Calculated vdW interaction energy (A) and electrostatic interaction energy (B) between two CuO NPs under varying concentrations of citric acid (0, 1, 10, and 100 mg/L).

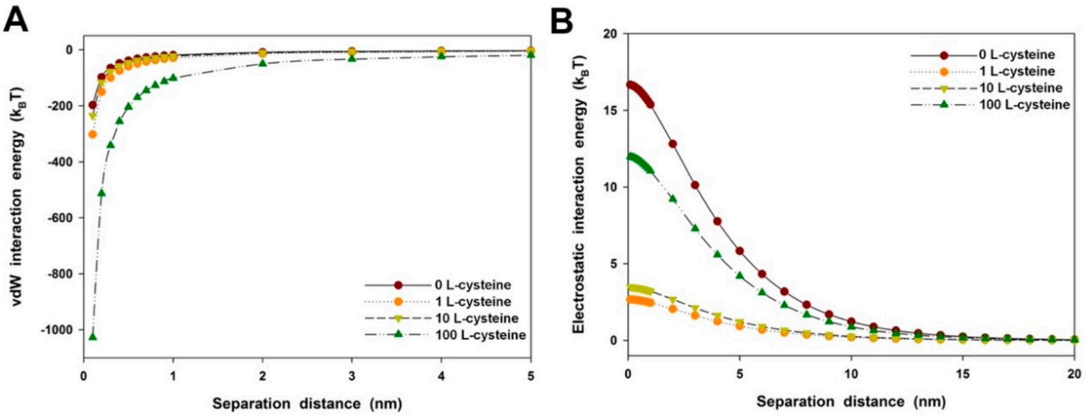


**Figure S7.** Calculated vdW interaction energy (A) and electrostatic interaction energy (B) between two CuO NPs under varying concentrations of L-cysteine (0, 1, 10, and 100 mg/L).

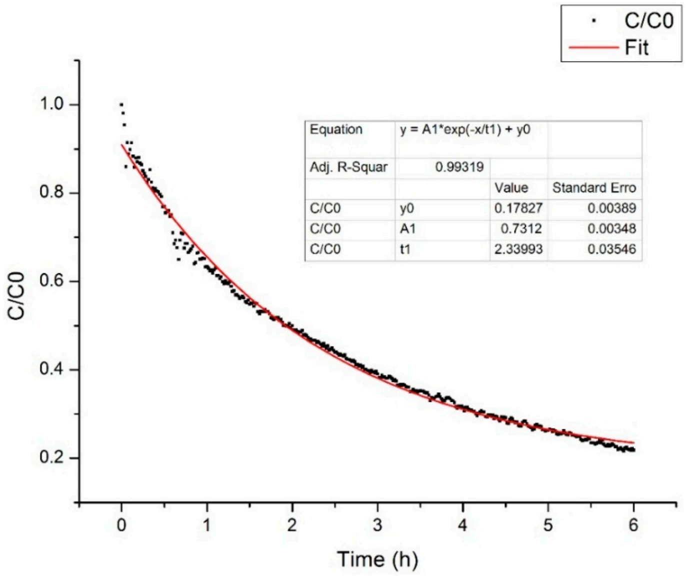


**Figure S8.** Fit of the sedimentation data to the Stokes equation. Mechanistic model for 100 mg/L 40 nm CuO NPs at pH 7 with 10 mM ionic strength.
